# Robot-assisted rehabilitation supports cortical network reorganization after nerve transfer surgery to treat chronic, complete cervical spinal cord injury

**DOI:** 10.64898/2026.05.26.26353736

**Published:** 2026-06-02

**Authors:** Amanda Bernstein, Justin M. Brown, Kathleen Friel, Edmund Hollis

## Abstract

Recovery of hand and arm function is critical for improving quality of life in individuals with tetraplegia due to spinal cord injury (SCI). Nerve transfer procedures can restore meaningful hand and arm function in chronic SCI, yet postoperative outcomes vary widely. We conducted a prospective, single-arm, open-label trial to assess the impact of intensive, robot-assisted rehabilitation training on functional recovery and cortical reorganization following nerve transfer. The primary endpoint was assessment of hand and arm function measured by the Box and Blocks Test.

We report the results from three participants, AIS A at enrollment, who completed six weeks of intensive robotic training at least 1 year after nerve transfer surgery ( NCT04041063 ). All participants demonstrated minimally important difference improvements in at least one secondary clinical outcome. These improvements were accompanied by cortical reorganization measured by transcranial magnetic stimulation motor mapping, indicating integration of the newly established peripheral motor pathways. No serious adverse events related to surgery or rehabilitation occurred.

Although recruitment was limited by the COVID-19 pandemic and precludes definitive conclusions regarding efficacy, these findings suggest that standardized, intensive robotic rehabilitation may enhance functional outcomes after nerve transfer surgery for chronic tetraplegia.

## Full Text Availability

The license terms selected by the author(s) for this preprint version do not permit archiving in PMC. The full text is available from the preprint server.

